# Case Report: Successful treatment of spondyloenchondrodysplasia with immune dysregulation using tofacitinib and ruxolitinib: a report of two pediatric cases

**DOI:** 10.3389/fphar.2025.1588003

**Published:** 2025-07-25

**Authors:** Chengzhu Liu, Zhiwei Xie, Min Wang, Jinhua Chu, Linhai Yang, Kunlong Zhang, Lingling Huang, Songji Tu, Huaju Cai, Zhengyu Wu, Liyuan Wang, Ningling Wang

**Affiliations:** ^1^Department of Pediatric Hematology and Oncology, The Second Affiliated Hospital of Anhui Medical University, Hefei, China; ^2^Department of Hematology and Oncology, Anhui Provincial Children’s Hospital, Hefei, China

**Keywords:** spondyloenchondrodysplasia, ACP5, case report, genetic disease, pediatric

## Abstract

Spondyloenchondrodysplasia with immune dysregulation (SPENCDI) is autosomal recessive hereditary disease caused by tartrate resistant acid phosphatase 5 (ACP5) mutations. The symptoms mainly involve the bone, immune system and nervous system, and the typical manifestations are short stature, abnormal development of long diaphyseal epiphysis, flat vertebra, and prone to various autoimmune diseases. Some children have muscle spasm, mild mental retardation, intracranial calcification and other neurological manifestations. Here we reported two cases of SPENCDI caused by a new mutation in ACP5. The clinical manifestations were autoimmune hemolytic anemia, immune thrombocytopenia, abnormal bone development, intracranial calcification, short stature, and growth retardation. The patients were girls and diagnosed with SPENCDI by genetic test.

## Introduction

Spondyloenchondrodysplasia with immune dysregulation (SPENCDI) is caused by biallelic mutations in the ACP5 gene, located on chromosome 19p13.2, which encodes tartrate-resistant acid phosphatase (TRAP) (Briggs et al., 2011; Lausch et al., 2011). These mutations result in the loss of regulation of TRAP activity on the function of osteopontin (OPN), leading to an excess of phosphorylated OPN. This excess promotes bone resorption through the activation of osteoclasts and causes immune dysregulation by stimulating type I interferon production (Cheng and Anderson, 2012).

This report describes two cases of SPENCDI. Case 1 was diagnosed at the Second Affiliated Hospital of Anhui Medical University, while Case 2 was treated at Anhui Provincial Children’s Hospital. A review of relevant literature was conducted to enhance physicians’ understanding of the disease and to reduce the rates of missed and misdiagnoses.

## Case description

**FIGURE 1:**
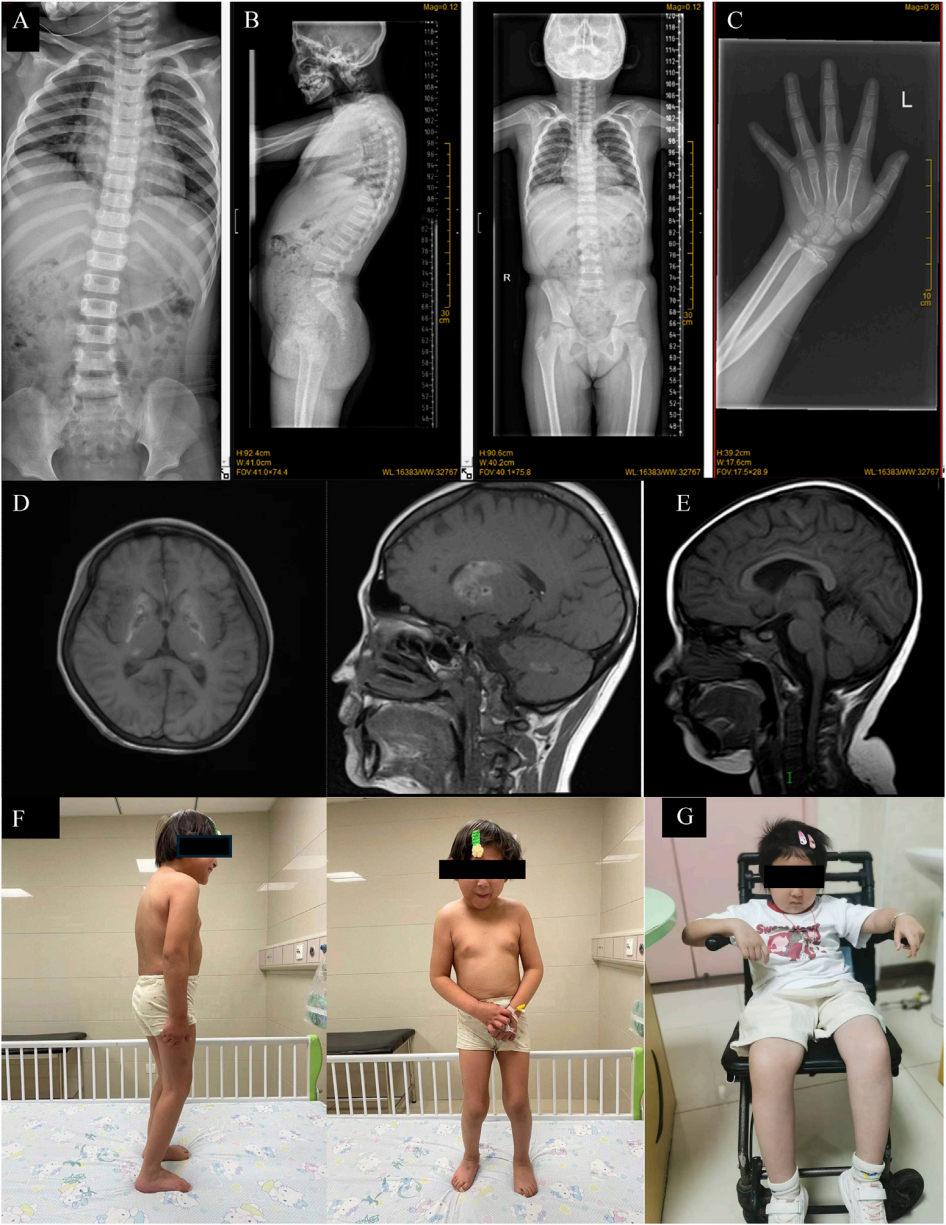
**(A)** X-Ray of spine of Case2 showing platyspondyly. **(B)** X-Ray of spine of Case 1 showing platyspondyly. **(C)** X-Ray of forearm and wrist of Case 1 showing metaphyseal dysplasia. **(D)** Brain MRI of Case 1 showed the formation of calcification foci in bilateral basal ganglia. **(E)** Brain MRI of Case 2 showed short T1 signal in bilateral pallidus and high T1WI signal in adenohypophysis. **(F)** Clinical photos of Case 1, standing relatively reluctantly; **(G)** Clinical photos of Case 2, in which the patient had been unable to stand on his own since the beginning.

Case 1 is a 13-year-old Chinese girl whose non-consanguineous parents are in good health. In February 2021, she was admitted to our hospital due to tea-colored urine and a dark yellow complexion without an identifiable cause. Physical examination revealed multiple brown pigmented spots on her face, a soft liver palpated 5 cm below the costal margin without tenderness, a spleen not palpable, both thumbs displaying adduction deformity, increased limb muscle tone, grade V muscle strength in both upper limbs, and negative pathological signs (Figure 1F). Her height was 116 cm (<−3 SD) and weight was 25.6 kg (<−3 SD). Laboratory results showed: hemoglobin (Hb) 51 g/L, reticulocyte percentage (Ret%) 24.38%, reticulocyte absolute count (Ret#) 0.339 × 10^12/L, lactate dehydrogenase (LDH) 3449 U/L, total bilirubin 59.5 μmol/L (indirect 55.0 μmol/L, direct 4.5 μmol/L), direct anti-human globulin test (Coombs) positive (Table 1), free triiodothyronine 2.550 pmol/L, free thyroxine 11.880 pmol/L, thyroid-stimulating hormone (TSH) 2.090 mIU/L, thyroid globulin antibody 157.00 IU/mL, and thyroid peroxidase antibody 84.38 IU/mL. Ferritin was 1660.0 μg/L, EBV-DNA 1.7 × 10^4 copies/mL, IGF-1 114.00 ng/mL (<−2 SD), and IGFBP-3 3.4 μg/mL (<−2 SD). X-Ray of spine showed platyspondyly and X-Ray of forearm and wrist showed metaphyseal dysplasia (Figures 1B,C). A brain MRI revealed symmetrical abnormalities in the bilateral basal ganglia (Figure 1D). Additional laboratory tests for this patient can be found in the Supplementary Material.

**TABLE 1:** Laboratory examination results of two children.

Laboratory tests	Case 1		Case 2		Laboratory normal reference range
	At first visit	6 months after ruxolitinib	At first visit	6 months after tofacitinib	
WBC (×10^9^/L)	3.31	2.56	11.7	4.26	3.50–9.50
HB (g/L)	51	117	104	147	115–150
PLT (×10^9^/L)	251	205	8	159	125–350
ALT (U/L)	28	36	22	16.9	7–40
AST (U/L)	263	37	74	31.1	13–35
TB (μmol/L)	59.5	7.7	8.9	6.5	0–21.0
DB (μmol/L)	4.5	2.4	3.3	2.8	0–4.0
IB (μmol/L)	55.0	5.3	5.6	3.7	0–17.0
LDH (U/L)	3,449	236	1572	156	120–250
BUN (μmol/L)	5.81	4.39	7.2	4	2.60–7.50
Cr (μmol/L)	50	28	38.4	27.1	41–73
Coombs	IgG (+), C3 (+)	(−)	IgG (+)	(−)	(−)
IgG	18.22	9.36	11.02	7.16	7.00–16.60
IgM	0.5	0.32	1.36	1.77	0.50–2.60
IgA	4.78	1.48	3.81	1.75	0.7–3.50
C3	1.15	1.29	1.1	1.02	0.83–0.77
C4	0.29	0.39	0.17	0.28	0.16–0.38
ANA*	(−)	(−)	(−)	(−)	(−)
T% (#/μL)	59.70% (377)	77.10% (738.00)	68.5% (646)	53.2% (875)	56%–86% (723–2737)
CD4^+^T% (#/μL)	7.80% (49.00)	14.70% (141.00)	10.7% (101)	21.7% (358)	33%–58% (404–1612)
CD8^+^T% (#/μL)	50.90% (321.00)	58.50% (561.00)	44.7% (422)	27.7% (456)	13%–39% (220–1129)
B% (#/μL)	38.10% (332.00)	14.70% (143.00)	28.2% (266)	35.1% (578)	5%–22% (80-616)
NK% (#/μL)	2.80% (24.00)	5.4% (52.00)	1.2% (12.00)	10.1% (167)	2.90% (80–724)

Note: *including: ANA, anti-U1RNP; anti-Sm, anti-SSA; anti-Ro52, anti-SSB; anti-Scl70, PM-Scl, anti-Jo1, CENP-B, anti-PCNA, anti-dsDNA, AnuA, AHA, anti-rRNP, and AMA-M2.

The initial diagnoses included autoimmune hemolytic anemia, skeletal dysplasia, intracranial calcification, short stature, and growth retardation. Genetic testing revealed an ACP5 c.464delT frameshift mutation in the patient’s peripheral blood DNA. According to the American College of Medical Genetics and Genomics (ACMG) criteria for classifying the clinical significance of genetic variants (Richards et al., 2015), this mutation is considered a “pathogenic variant” and was not previously reported in the HGMD database. The patient was ultimately diagnosed with SPENCDI, and her treatment regimen included human immunoglobulin (20 g for 2 days), methylprednisolone (500 mg for 3 days, reduced to 50 mg for 6 days), followed by prednisone (15 mg three times daily), which was gradually tapered to 10 mg daily. By July 2021, the Coombs test was negative. However, on 13 December 2021, Coombs test turned positive again, and there was no clinical symptoms such as jaundice during the follow-up visit. The child’s parents chose to continue oral prednisone treatment. Considering the long history of oral glucocorticoid treatment and the poor control effect of AIHA, oral sirolimus was added on 17 April 2023, but Coombs test did not recover to negative. On 16 August 2024, oral ruxolitinib (5 mg daily) combined with prednisone (7.5 mg daily) was started. Coombs test successfully turned negative after 1 month of treatment adjustment. The patient is currently being monitored regularly.

Case 2 involves an 8-year-old girl, also from non-consanguineous healthy parents. In June 2017, when she was 1 year old, skin ecchymosis was the first symptom. Physical examination revealed scattered ecchymoses and hemorrhagic spots across her body. She had decreased muscle strength in her upper limbs (grade IV) and lower limbs (grade III), could sit independently but could not crawl or stand (Figure 1G). X-ray of spine also showed platyspondyly (Figure 1A). Her weight and height were within normal limits. Laboratory results showed: Hb 104 g/L, platelet count 2 × 10^9/L, CD3^+^CD4^+^ cells 10.7%, CD3^+^CD4^+^ cell count 101/μL, CD3^+^CD8^+^ cells 44.7%, and CD3^+^CD8^+^ cell count 422/μL. Biochemical indices, including liver and kidney function tests, were normal (Table 1).

The initial diagnosis was autoimmune thrombocytopenia. From July 2017 to September 2020, she was hospitalized several times for glucocorticoid and immunoglobulin shock therapy, followed by long-term oral prednisone maintenance therapy. On 22 March 2022, she presented with sudden onset of yellowish skin, Hb of 38 g/L, total bilirubin 84.5 μmol/L (indirect 51.4 μmol/L, direct 33.1 μmol/L) and a positive Coombs test, leading to a diagnosis of autoimmune hemolytic anemia. Consequently, glucocorticoid and human immunoglobulin shock therapies were re-administered, but hemoglobin levels failed to improve significantly. The patient received four doses of CD20 monoclonal antibody (100 mg once a week) in June 2022, accompanied by mycophenolate mofetil (750 mg daily). By August 2022, her hemoglobin had recovered to over 90 g/L, and the bilirubin level returned to normal. To confirm the diagnosis further, genetic testing was conducted, revealing an ACP5 mutation (Table 2). The patient was ultimately diagnosed with SPENCDI and was switched to oral Tofacitinib (5 mg twice daily) in April 2023, with regular follow-ups. Currently, her hemoglobin and platelet levels remain within the normal range.

**TABLE 2:** Gene mutation information of the patients.

Case	Gene	Reference transcript	Position	cDNA level	Protein level	Status	Father	Mother
1	ACP5	NM_001111035	Exon6	c.464delT	p. Leu155fs	Heterozygous	No	Heterozygous carrier
	ACP5	NM_001111035	Exon4	c.229G>C	p. Gly77Arg	Heterozygous	Heterozygous carrier	No
2	ACP5	NM_001111035	Exon7	c.740T>C	p. Leu247Pro	Heterozygous	No	Heterozygous carrier
	ACP5	NM_001111035	Chromosome 4–7 heterozygosity is missing					

## Discussion and conclusion

SPENCDI is a rare complex autosomal recessive skeletal dysplasia characterized by platyspondyly, enchondroma-like radiolucent metaphyseal lesions, dysplasia of the metaphyses in long bones, and a commonly observed short-trunked stature, along with various extra-skeletal presentations (Utsumi et al., 2017). The signs and symptoms of SPENCDI can manifest at any point from infancy to adolescence. In addition to familial variability, the onset, severity, and clinical presentation of the disease may vary (Navarro et al., 2008). However, Zhong et al. reported that skeletal abnormalities are the most frequent manifestation, followed by autoimmune diseases and involvement of the central nervous system (Zhong et al., 2018). In this study, the patients reported exhibited typical symptoms such as immune hemolytic anemia, immune thrombocytopenia, skeletal dysplasia, intracranial calcification, short stature, and growth retardation. These symptoms are consistent with the known clinical presentation of SPENCDI, but also highlight the clinical diversity and complexity of the condition. In particular, autoimmune hemolytic anemia and immune thrombocytopenia are challenging to diagnose.

In patients with SPENCDI, skeletal abnormalities consist of flattening of spinal bones, abnormalities at the ends of long limb bones, and lesions in long and spinal bones observable through X-rays. Additional skeletal issues arise due to abnormalities in the resilient, flexible cartilaginous tissue that comprises the skeleton during early development. Patients with SPENCDI often exhibit areas where cartilage cannot transition into bone, as well as non-cancerous growths of cartilage. These skeletal and cartilaginous complications contribute to the short stature characteristic of SPENCDI patients (Al Kaissi et al., 2013; Robinson et al., 1991). A review of the literature revealed that the reported skeletal phenotypes of SPENCDI patients exhibited a wide range—ranging from normal to −6.5 SD in height (Al-Kateb et al., 2024). In some patients, the severity of short stature significantly increased with age, with varying types of short stature being identified between short trunk and short limb presentations. The response to growth hormone therapy in patients with skeletal dysplasia remains contentious; however, further examination of the genes involved in bone formation may illuminate which skeletal dysplasias respond favorably to growth hormone treatment.

An overactive immune system can lead to a heightened risk of autoimmune diseases and impair the body’s ordinary immune response to harmful invaders, resulting in frequent infections. Immune system dysfunction can provoke a variety of disorders such as thrombocytopenia, hemolytic anemia, hypothyroidism, or chronic inflammatory conditions like systemic lupus erythematosus and rheumatoid arthritis (An et al., 2017). Some individuals with SPENCDI also experience neurological complications, including muscle spasms, coordination difficulties (ataxia), and intellectual disabilities. Additionally, abnormal calcium deposits (calcifications) in the brain may occur (Elhossini et al., 2023). The mechanisms responsible for other aspects of SPENCDI, such as dyskinesia and intellectual disability, remain unclear. Patients with SPENCDI typically have a reduced life expectancy due to a range of immune system complications, although the extent of this variation is significant. However, the specific mechanism of ACP5 mutation and immunomodulatory function is not fully understood, so further basic research is needed to explore this association.

In SPENCDI, excessive production of type I interferons can trigger the release of nuclear antigens from dying cells and the maturation of autoreactive B cells, ultimately leading to autoimmune disease (Briggs et al., 2011; Cheng and Anderson, 2012). A comprehensive survey involving 26 SPENCDI patients found that 85% exhibited at least one autoimmune manifestation (Briggs et al., 2016). Both type I interferons bind to specific receptors comprising the IFNAR1 and IFNAR2 subunits. When interferon binds to its receptor, IFNAR1 and IFNAR2 are activated, dimerize, and form unique transmembrane protein complexes that activate downstream signaling pathways. Activated IFNAR1 interacts with TYK2, and IFNAR2 interacts with JAK1 to initiate STATs, MAPK, and PI3K signaling pathways, producing various biological effects. Although the use of JAK inhibitors (Ruxolitinib and Tofacitinib) in our cases showed promising results, further studies are needed to determine the optimal treatment protocols. These treatments showed improvement in hemoglobin and platelet counts, but the long-term effects on bone growth and immune regulation remain unclear.

A case report by Akshaya Chougule et al. demonstrated that an oral JAK1/2 inhibitor improved the patient’s clinical symptoms (Chougule et al., 2023). Yael Gernez et al. also reported two patients with refractory Evans syndrome with SPENCDI who were successfully treated with JAK1/JAK2 inhibitors (Gernez et al., 2024). And in our study, Case 1 has been receiving Ruxolitinib 5 mg daily since 14 August 2024, with prednisone adjusted to 7.5 mg daily. Currently, complete blood counts, liver function tests, renal function tests, triglycerides, DAT tests, and blood glucose levels are monitored weekly, with the goal of tapering off prednisone as soon as possible. In Case 2, treatment with oral Tofacitinib has maintained normal hemoglobin and platelet counts. Although case reports suggest that early initiation and prolonged use of JAK1/2 inhibitors may help prevent neurological complications and enhance quality of life, further trials in larger cohorts are necessary to establish the optimal dosage, efficacy, and side effect profiles of JAK1/2 inhibitors in SPENCDI. Further studies should focus on the role of JAK inhibitors in the management of SPENCDI, with larger cohorts to explore dose optimization and long-term outcomes. Additionally, genetic research into other variants of ACP5 and related immune pathways may provide new insights into potential therapeutic targets.

## Data Availability

The original contributions presented in the study are included in the article/Supplementary Material, further inquiries can be directed to the corresponding author.

## Abbreviations

SPENCDI, Spondyloenchondrodysplasia with immune dysregulation; ACP5, acid phosphatase 5; TRAP, tartrate-resistant acid phosphatase; OPN, osteopontin; WBC, white blood cell; HB, haemoglobin; PLT, Platelets; ALT, Alanine Aminotransferase; AST, Aspartate Aminotransferase; TB, Total Bilirubin; DB, Direct Bilirubin; IB, Indirect Bilirubin; LDH, Lactate Dehydrogenase; BUN, Blood Urea Nitrogen; Cr, Creatinine.

## References

[B1] Al Kaissi A. Ben Chehida F. Ben Ghachem M. Klaushofer K. Grill F. (2013). Dysmorphic facies and diffuse posterior spine ankylosis in a patient with unusual form of spondyloenchondrodysplasia (spranger type IV). Eur. Spine J. 22 (Suppl. 3), S409–S415. 10.1007/s00586-012-2518-2 23053755 PMC3641239

[B2] Al-Kateb F. Dyab D. Almadani B. Al-Enezi N. (2024). Spondyloenchondrodysplasia with immune dysregulation, but without skeletal dysplasia, in a six-year-old boy: a case report. Cureus 16 (5), e60314. 10.7759/cureus.60314 38883133 PMC11177273

[B3] An J. Briggs T. A. Dumax-Vorzet A. Alarcón-Riquelme M. E. Belot A. Beresford M. (2017). Tartrate-resistant acid phosphatase deficiency in the predisposition to systemic lupus erythematosus. Arthritis Rheumatol. 69 (1), 131–142. 10.1002/art.39810 27390188

[B4] Briggs T. A. Rice G. I. Adib N. Ades L. Barete S. Baskar K. (2016). Spondyloenchondrodysplasia due to mutations in ACP5: a comprehensive survey. J. Clin. Immunol. 36 (3), 220–234. 10.1007/s10875-016-0252-y 26951490 PMC4792361

[B5] Briggs T. A. Rice G. I. Daly S. Urquhart J. Gornall H. Bader-Meunier B. (2011). Tartrate-resistant acid phosphatase deficiency causes a bone dysplasia with autoimmunity and a type I interferon expression signature. Nat. Genet. 43 (2), 127–131. 10.1038/ng.748 21217755 PMC3030921

[B6] Cheng M. H. Anderson M. S. (2012). Monogenic autoimmunity. Annu. Rev. Immunol. 30, 393–427. 10.1146/annurev-immunol-020711-074953 22224765 PMC6249029

[B7] Chougule A. Taur P. Gowri V. Consortium C. O. E. Desai M. M. (2023). SPENCD presenting with evans phenotype and clinical response to JAK1/2 Inhibitors-a report of 2 cases. J. Clin. Immunol. 43 (2), 331–334. 10.1007/s10875-022-01400-8 36376765

[B8] Elhossini R. M. Elbendary H. M. Rafat K. Ghorab R. M. Abdel-Hamid M. S. (2023). Spondyloenchondrodysplasia in five new patients: identification of three novel ACP5 variants with variable neurological presentations. Mol. Genet. Genomics 298 (3), 709–720. 10.1007/s00438-023-02009-1 37010587 PMC10133048

[B9] Gernez Y. Narula M. Cepika A. M. Valdes Camacho J. Hoyte E. G. Mouradian K. (2024). Case report: refractory evans syndrome in two patients with spondyloenchondrodysplasia with immune dysregulation treated successfully with JAK1/JAK2 inhibition. Front. Immunol. 14, 1328005. 10.3389/fimmu.2023.1328005 38347954 PMC10859398

[B10] Lausch E. Janecke A. Bros M. Trojandt S. Alanay Y. De Laet C. (2011). Genetic deficiency of tartrate-resistant acid phosphatase associated with skeletal dysplasia, cerebral calcifications and autoimmunity. Nat. Genet. 43 (2), 132–137. 10.1038/ng.749 21217752

[B11] Navarro V. Scott C. Briggs T. A. Barete S. Frances C. Lebon P. (2008). Two further cases of spondyloenchondrodysplasia (SPENCD) with immune dysregulation. Am. J. Med. Genet. A 146A (21), 2810–2815. 10.1002/ajmg.a.32518 18924170

[B12] Richards S. Aziz N. Bale S. Bick D. Das S. Gastier-Foster J. (2015). Standards and guidelines for the interpretation of sequence variants: a joint consensus recommendation of the American college of medical genetics and genomics and the association for molecular pathology. Genet. Med. 17 (5), 405–424. 10.1038/gim.2015.30 25741868 PMC4544753

[B13] Robinson D. Tieder M. Copeliovitch L. Halperin N. (1991). Spondyloenchondrodysplasia. A rare cause of short-trunk syndrome. Acta Orthop. Scand. 62 (4), 375–378. 10.3109/17453679108994474 1882681

[B14] Utsumi T. Okada S. Izawa K. Honda Y. Nishimura G. Nishikomori R. (2017). A case with spondyloenchondrodysplasia treated with growth hormone. Front. Endocrinol. (Lausanne) 8, 157. 10.3389/fendo.2017.00157 28740483 PMC5502255

[B15] Zhong L. Q. Wang L. Song H. M. Wang W. Wei M. He Y. Y. (2018). Zhonghua Er Ke Za zhi. 56, 611–616. 10.3760/cma.j.issn.0578-1310.2018.08.011 30078244

