# Supplementary material for: Case Report: Successful treatment of spondyloenchondrodysplasia with immune dysregulation using tofacitinib and ruxolitinib: a report of two pediatric cases
Source: Front Pharmacol. 2025 Jul 25;16:1588003. doi: 10.3389/fphar.2025.1588003 (PMC12331710; doi:10.3389/fphar.2025.1588003)
Supplement: Supplementary file 1 [file DataSheet1.docx]

Supplementary Material

Successful Treatment of spondyloenchondrodysplasia with Immune Dysregulation Using Tofacitinib and Ruxolitinib: A Report of Two Pediatric Cases

**Chengzhu Liu^1†^, Zhiwei Xie ^1†^, Min Wang^2†^, Jinhua Chu^1^, Linhai Yang ^1^, Kunlong Zhang ^1^, Lingling Huang^1^, Songji Tu^1^, Huaju Cai^1^, Zhengyu Wu^1^, Anbang Wei^1^, Liyuan Wang^1^, Ningling Wang^1*^**

*** Correspondence:**Ningling Wang
[zwnltt@126.com](mailto:zwnltt@126.com)

# Supplementary Table

# GH stimulation test (at the age of 13 years) of Case1

| **Levodopa tolerance test** | **Basal** | **30 min** | **60 min** | **90 min** | **120 min** |
| --- | --- | --- | --- | --- | --- |
| GH (ng/mL) | 1.43 | 1.86 | 3.06 | 5.10 | 4.31 |

Biochemical/Hormonal data (at the age of 13 years)

| **Parameters** | **Patient1** |
| --- | --- |
| Ca (mmol/L) | 1.88 |
| AST (IU/L) | 263 |
| ALT (IU/L) | 28 |
| TG (mmol/L) | 2.53 |
| LDH (IU/L) | 3449 |
| CK-MB (IU/L) | 210 |
| IGF-I (ng/mL) | 114.00 |
| IGFBP-3 (μg/mL) | 3.4 |
| TSH (mIU/mL) | 2.090 |
| FT4 (pmol/L) | 11.880 |
| FT3 (pmol/L) | 2.55 |
| Ferritin (μg/L) | 1660.0 |
| ACTH (pg/ml) | <5.0 |
| β-HCG (mIU/mL) | <0.1 |

# Supplementary Figure


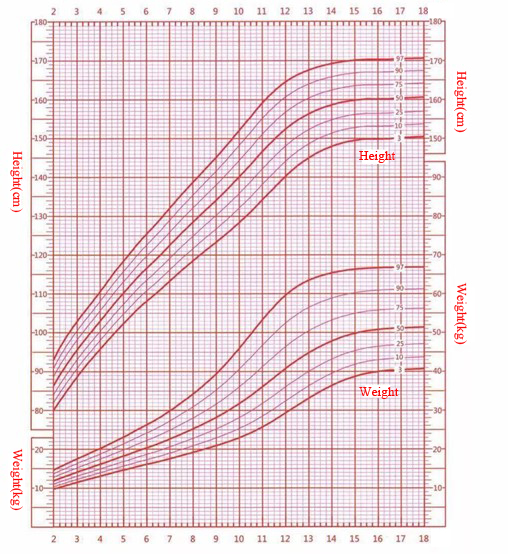


**Supplementary Figure.** Height and weight percentile of Chinese girls aged 2-18. The Case1: Height: 116cm (< -3SD); Weight: 25.6kg (< -3SD)
